# In Silico Identification of Rosmarinic Acid as a Putative Natural Product Binder of the PI3Kα Allosteric Site

**DOI:** 10.3390/biology15211935

**Published:** 2026-10-25

**Authors:** Wenqing Jia, Yukui Ma, Xianchao Cheng, Shuyu Luo

**Affiliations:** 1 School of Chemistry and Chemical Engineering, Qilu Normal University, Jinan 250200, China; 2 Tianjin Key Laboratory on Technologies Enabling Development of Clinical Therapeutics and Diagnostics (Theranostics), School of Pharmacy, Tianjin Medical University, Tianjin 300070, China; 3 Department of Oral Histology and Pathology, School of Stomatology, Tianjin Medical University, Tianjin 300070, China; 4 Tianjin Key Laboratory of Oral Soft and Hard Tissues Restoration and Regeneration, Tianjin 300070, China

**Keywords:** *PIK3CA*, allosteric PI3Kα inhibitors, tumor, natural product

## Abstract

*PIK3CA* mutations drive tumorigenesis by activating phosphatidylinositol 3-kinase alpha (PI3Kα). The allosteric inhibitor STX-478 exhibits high selectivity toward PI3Kα and avoids adverse effects such as hyperglycemia. Hyperglycemia is the main reason for the limited clinical application of ATP-competitive PI3Kα inhibitors. To date, no allosteric PI3Kα inhibitors have been approved for clinical use. Consequently, developing novel allosteric PI3Kα inhibitors represents a promising therapeutic strategy for patients harboring *PIK3CA* mutations. This study establishes a workflow for discovering computationally prioritized allosteric PI3Kα inhibitor candidates, integrating virtual screening, multi-platform druggability evaluation, molecular dynamics simulations, metabolic pathways and metabolites analysis. We used virtual screening to identify computationally prioritized allosteric PI3Kα inhibitor candidates from a small molecule library based on the binding mode between STX-478 and PI3Kα, and further filtered compounds with favorable drug-like properties. Molecular dynamics simulations were carried out to assess the binding stability of these candidates with the protein. We also characterized the metabolic profile of the hit compound. Rosmarinic acid (an ester of caffeic acid and 3,4-dihydroxyphenyllactic acid compound) was screened out as a computationally prioritized allosteric PI3Kα inhibitor candidate. The CDOCKER energy of rosmarinic acid (−69.4529 kcal/mol) in complex with PI3Kα was lower than that of STX-478 (−66.7128 kcal/mol). Molecular dynamics simulations revealed that rosmarinic acid can stably bind to PI3Kα. ADMET prediction suggested that rosmarinic acid possesses favorable safety profiles and acceptable pharmacokinetic properties. This study lays a computational foundation for screening potential allosteric PI3Kα inhibitor candidates.

## Simple Summary

*PIK3CA* is frequently overexpressed and mutated in tumor tissues, which causes aberrant activation of PI3Kα and thereby drives tumorigenesis and progression. A key advantage of the allosteric PI3Kα inhibitor STX-478 for tumor therapy is its ability to avoid metabolic dysfunction, a major drawback of ATP-competitive PI3Kα inhibitors. To facilitate the discovery of similar agents, we combined virtual screening with multiple computational functional platforms based on the binding mode between STX-478 and PI3Kα to rapidly identify predicted ligands targeting the PI3Kα allosteric pocket. This study reports a computationally prioritized allosteric PI3Kα inhibitor candidate, rosmarinic acid, and provides valuable information for further discovery of ligands targeting the PI3Kα allosteric pocket.

## 1. Introduction

**Figure 1:**
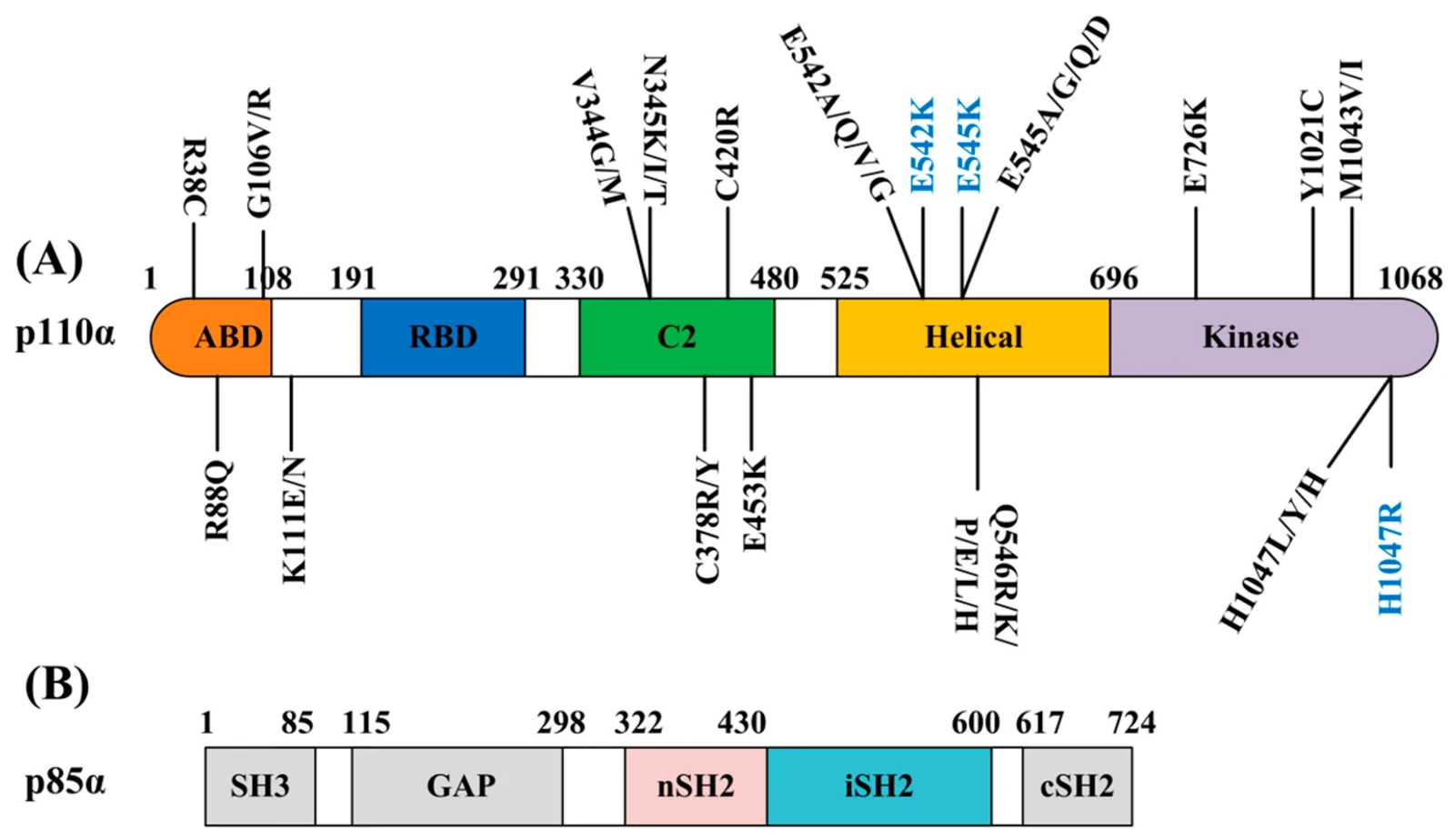
(**A**) The structure of p110α and mutation sites. Three most common hotspot mutations are marked in blue font; black font represents other mutation sites. (**B**) p85α structure.

Phosphoinositide 3-kinases (PI3Ks) represent an evolutionarily well-conserved family of lipid kinases that are widely expressed in mammalian cells. PI3Ks can convert extracellular signals such as growth factors and cytokines into intracellular signals, thereby influencing biological functions [1,2,3,4]. Research has shown that PI3Kα is abnormally activated in various solid tumors and is implicated in the progression of cancers such as head and neck cancer, endometrial cancer, gastric cancer, and colon cancer [5]. Structurally, PI3Kα comprises a catalytic subunit (p110α) paired with a regulatory subunit (p85α) [6,7]. p85α tightly binds to the catalytic subunit via its iSH2 domain, suppressing p110α’s activity in the resting state. When the nSH2 domain interacts with phosphorylated tyrosine residues on activated receptors, p110α is released from inhibition and can exert its biological functions (Figure 1) [7].

The abnormal activation of PI3Kα occurs in nearly 50% of malignant tumors [8]. It affects the proliferation, migration, invasion, and angiogenesis of tumor cells. Abnormal activation of pathways can also lead to drug resistance and generate an immunosuppressive tumor microenvironment [3]. *PIK3CA* encodes the catalytic subunit p110α of the PI3Kα, and its mutation leads to abnormal activation of PI3Kα, which is the most common self-activation mode [7]. *PIK3CA* gene mutation is a functional acquired mutation that leads to uncontrolled growth, proliferation, and survival of cells. From the perspective of protein structure, there are many *PIK3CA* mutation sites, among which 63% of mutations occur in E542 and E545 (helical domain) and H1047 (kinase domain). In most cases, exon 9 harbors mutations at positions E542 and E545, where both residues are replaced by LYS, which can eliminate the inhibitory interactions between the N-terminal SH2 domains of p110α and p85α regulatory subunits [9,10,11]. H1047 mainly mutates into ARG on exon 20, which frequently occurs in cancer and may enhance the activity of p110α by affecting the binding of PIP2 to lipid membranes [12]. Safe and effective PI3Kα inhibitors are key to improving the quality of life for cancer patients.

ATP-competitive PI3Kα inhibitors have been widely reported, and both FDA-approved PI3Kα inhibitors belong to this class. Alpelisib, a PI3Kα inhibitor, has obvious inhibitory effect on wild-type and mutant PI3Kα and can be used to treat patients with advanced or metastatic breast cancer that carries *PIK3CA* mutations, accompanied by hyperglycemia, drug resistance and other side effects in clinical trials [13]. As a dual-acting agent, inavolisib acts as a potent PI3Kα inhibitor and a mutant PI3Kα degrader, and is administered to patients with locally advanced or metastatic solid tumors carrying *PIK3CA* mutations [14]. Copanlisib is a pan PI3K inhibitor administered intravenously, clinically used to treat recurrent follicular lymphoma (FL) [15]. In a phase II MATCH trial (NCT02465060), copanlisib exhibits favorable clinical therapeutic effects for refractory malignancies with *PIK3CA* mutations [16]. Compared with using CTLA-4 monoclonal antibody alone, the combination of copanlisib with paclitaxel and CTLA-4 monoclonal antibody can significantly inhibit PyMT growth and increase the activation of CD8+T cells in tumors [17]. CYH33, GDC-0084 and NVP-BEZ235 also show strong therapeutic efficacy in *PIK3CA*-mutant solid cancers [18,19,20,21,22,23].

Hyperglycemia stands as the most prevalent adverse effect of ATP-competitive PI3Kα inhibitors. Accumulating research has verified that the p110α catalytic subunit of PI3Kα serves as the core mediator of insulin signal transduction in cells across various pathological conditions, with malignant tumors being the most representative case. Small-molecule agents that selectively inhibit p110α are capable of blocking insulin-activated signaling cascades. Such inhibitory effect accelerates hepatic glycogen breakdown and suppresses glucose absorption in skeletal muscle and adipose tissues, which eventually triggers adverse hyperglycemic side effects [3]. Alpelisib (Piqray), the world’s first selective PI3Kα inhibitor to enter the market, was assessed in the phase III SOLAR-1 clinical trial. Within this trial, 6.3% of participants stopped drug administration due to hyperglycemia of any severity, and 4.2% terminated treatment specifically because of grade 3 or higher hyperglycemic adverse events [24]. Hyperglycemia of all grades emerged in 65% of patients receiving alpelisib, among whom 37% developed high-grade hyperglycemia classified as grade 3 or above [24]. The INAVO120 clinical investigation reported that, in 54 subjects with baseline fasting blood glucose (FBG) exceeding 160 mg/dL, 52 cases (accounting for 96%) achieved at least one-grade alleviation of glucose abnormalities following inavolisib intervention, with a median time of 8 days until symptom improvement [25].

Hyperglycemic adverse reactions triggered by alpelisib and inavolisib generally manifest within the initial month of medication administration. Regular and strict detection of FBG enables early identification of hyperglycemia, which is conventionally measured after an 8 to 12 h overnight fasting period prior to each drug dose. For alpelisib therapy, FBG testing is mandated once per week during the first two treatment weeks and shifts to monthly examinations thereafter, alongside quarterly glycated hemoglobin (HbA1c) measurements. Moreover, the SOLAR-1 trial amended its research protocol to incorporate a day 8 follow-up visit dedicated to hyperglycemic risk screening [25]. In line with the official U.S. prescribing information for inavolisib, clinicians need to evaluate FBG and HbA1c levels and adjust abnormal glucose indicators to the normal range prior to treatment initiation, and implement continuous long-term monitoring throughout medication [25].

Currently, allosteric PI3Kα inhibitors have also entered clinical studies. The PI3Kα inhibitory activity of STX-478 against H1047R site mutation is stronger than that of wild-type and E545K mutation, and it exhibits excellent anti-head and neck squamous cell carcinoma (HNSCC) effects in cell line-derived xenograft tumor models (CDX) and patient-derived xenograft tumor models (PDX), without causing metabolic dysfunction [26]. STX-478 can prevent systemic metabolic dysfunction induced by alpelisib at equivalent effective doses [26]. The phase II/III clinical trials of STX-478 for breast cancer and other solid tumors are also being recruited (NCT07174336, NCT05768139). RLY-2608 demonstrates superior inhibition against PI3Kα mutants. The ReDiscover phase I trial further demonstrated durable target inhibition in individuals suffering from *PIK3CA*-driven solid cancers, exhibiting minimal disruption to glucose homeostasis alongside acceptable safety and antitumor efficacy [27]. The phase III clinical trial of RLY-2608 for breast cancer is also being recruited (NCT06982521). In addition, clinical trials are also evaluating the efficacy of this drug for *PIK3CA* related overgrowth spectrum (PROS, NCT06789913, phase II). LOXO-783 achieves high selectivity for the PI3Kα H1047R mutant, showing promising therapeutic activity in breast cancer harboring this mutation (NCT05307705).

The PI3Kα inhibitors currently used in clinical trials are ATP-competitive inhibitors, exerting anti-tumor effects by targeting the ATP binding site of PI3Kα. Due to the high homology of ATP binding sites among different subtypes of the PI3K family, off-target effects occur, resulting in concentration-dependent serious adverse reactions such as hyperglycemia, rash, diarrhea, etc., which significantly affect the treatment efficacy, and have obvious clinical limitations [28,29]. However, there is relatively little research on allosteric inhibitors with low side effects and high PI3Kα inhibitory activity against *PIK3CA* mutations. To date, no allosteric PI3Kα inhibitors have been granted FDA approval, and developing new PI3Kα allosteric inhibitors is a new approach for cancer treatment.

In order to discover novel PI3Kα allosteric inhibitors with diverse structures, we previously modified the existing PI3Kα allosteric inhibitors STX-478 and RLY-2608 through scaffold hopping strategies [29,30]. We found that J-53 and H-18 could stably bind to the PI3Kα allosteric site, with J-53 having acceptable safety. The carbonyl group present in J-53 could form hydrogen bonds with ILE1019, making it more stable to bind to PI3Kα and contributing to its activity. In addition, the predicted LD_50_ of H-18 was 2000 mg/kg, much higher than RLY-2608 (300 mg/kg), indicating high safety.

In addition to directly modifying the structure of existing allosteric PI3Kα inhibitors, natural products also provide guarantees for the development of innovative drugs. Natural products have diverse structures and low toxicity. Their unique nuclear structure can also provide a structural basis for drug development, showing great potential in candidate drug research and accelerating the drug development process. About 30% of marketed drugs are derived from plant extracts or microbial metabolites, and nearly two-thirds of people worldwide have received treatment with natural products [31]. Natural products have become an important source of novel PI3Kα allosteric inhibitors. Research has shown that certain flavonoids, alkaloids, and other compounds have been found to effectively inhibit the activity of PI3Kα, while exhibiting low toxicity to normal cells [32]. Quercetin inhibited proliferation of HCT 116 and HT 29 cells, downregulating the mRNA expression of key genes (*PIK3CA*, *PIK3R1*, *AKT1*) in the PI3K-Akt pathway [33]. These natural products may selectively inhibit PI3Kα by binding to specific structural domains of PI3Kα, affecting the conformation of their active centers or their interactions with substrates.

**Figure 2:**
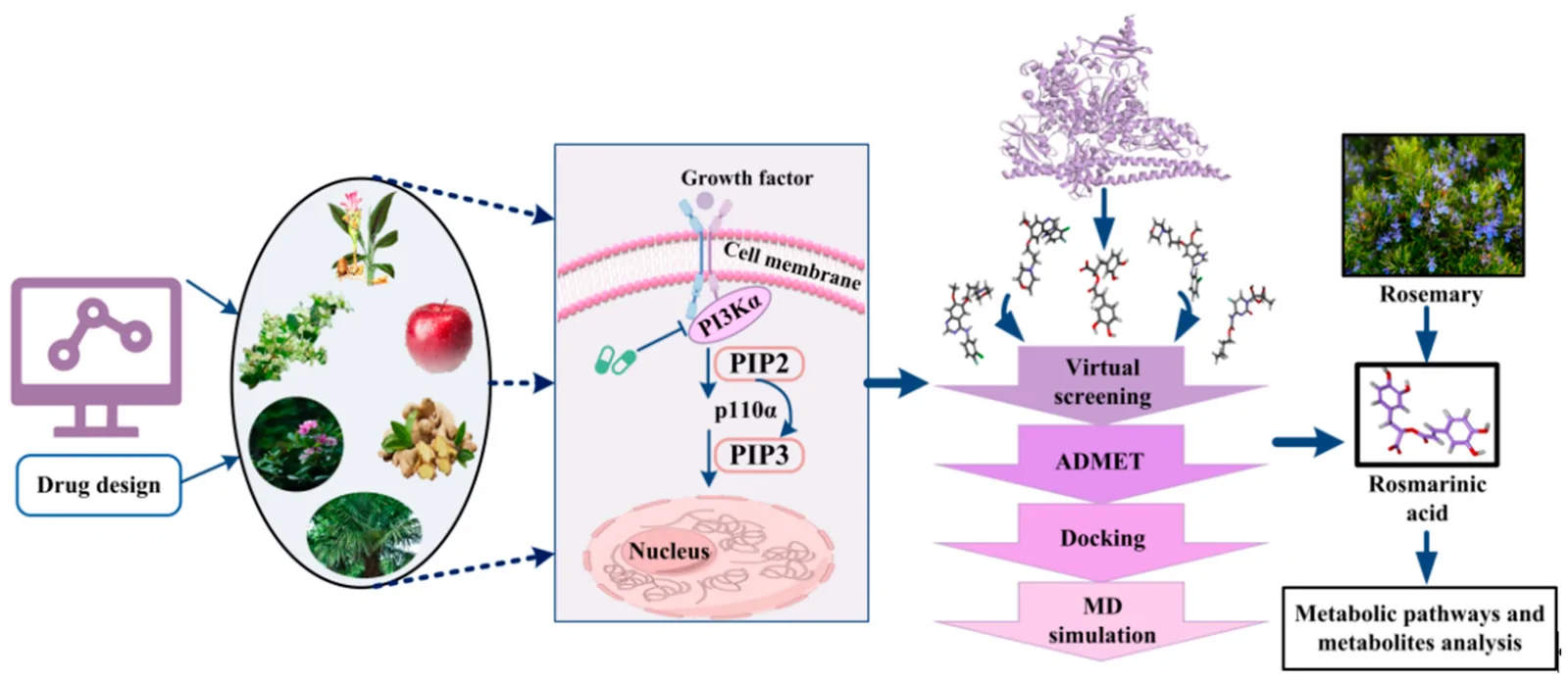
Virtual-screening-based identification of candidate ligands for the PI3Kα allosteric pocket.

To discover more novel PI3Kα allosteric inhibitors with good therapeutic effects, this study established a dedicated screening platform. We built a comprehensive screening system, including LibDock and CDOCKER-based molecular docking, multi-platform ADMET prediction (Discovery Studio, SwissADME, ADMETlab 3.0, ProTox 3.0), molecular dynamics simulations, metabolic pathways and metabolites analysis. Finally, the promising candidate rosmarinic acid was identified from a small molecule library (Figure 2).

As an ester of caffeic acid and 3,4-dihydroxyphenyllactic acid, rosmarinic acid possesses extensive antitumor, antioxidant, anti-inflammatory and antibacterial activities, thus holding great value for pharmacological and nutritional research. Gupta et al. verified that rosmarinic acid alleviates UVB-triggered mitochondrial damage and oxidative disorder closely linked to skin cancer progression by testing its impacts on primary human dermal fibroblasts and HaCaT keratinocytes [34]. Rosmarinic acid also exhibits promising therapeutic efficacy against various cancers including glioma, osteosarcoma, pancreatic cancer, liver cancer, colorectal cancer, breast cancer, oral cancer and lung cancer [35]. Previous reports have clarified that modulation of the PI3K/AKT/mTOR signaling pathway constitutes one of the primary mechanisms underlying its antitumor activity [36,37,38,39]. Our study indicates that rosmarinic acid is a computationally prioritized allosteric PI3Kα inhibitor candidate, which may modulate the PI3K/AKT/mTOR signaling pathway by targeting the allosteric site of PI3Kα.

## 2. Materials and Methods

### 2.1. Correlation Analysis Between PIK3CA and Tumor

Accumulating evidence from earlier investigations indicates that *PIK3CA* is closely correlated with the onset and progression of several malignancies, including endometrial cancer, colorectal cancer, HNSCC, and breast cancer. We used GeneCard (https://www.genecards.org/, accessed on 16 May 2025) to analyze the relationship between *PIK3CA* and the above tumors. We searched for endometrial cancer, colorectal cancer, HNSCC and breast cancer as keywords, and displayed the cancer-related gene results.

### 2.2. Virtual Screening and Molecule Docking Study

Virtual screening is an effective tool for drug discovery. This tool was employed for innovative drug discovery, targeting the PI3Kα (allosteric binding site, PDB ID: 8TGD) for systematic investigation [40]. LibDock in Discovery Studio 3.5 is a rigid receptor-based docking suite for fast and accurate screening. Virtual screening was carried out using the LibDock module implemented in Discovery Studio 3.5 on our in-house small molecule library. Prior to molecular docking, protein preparation was performed using Discovery Studio 3.5 to optimize the protein structure. Given the known active site, the “Define and Edit Binding Sites” function within Discovery Studio 3.5 was applied to precisely define the binding region. Subsequently, the “From Current Selection” option was adopted to generate a spherical binding pocket near major functional residues (LEU911, GLY912, LYS941, ARG949, GLU950, ASP1018, ILE1019, and ILE1022) within the allosteric site. The geometric parameters were optimized to a radius of 8.22 Å, with the center coordinates set at X = 19.22, Y = −28.87, and Z = 92.63. This predefined site-sphere was used for both LibDock high-throughput virtual screening and subsequent CDOCKER refinement. The library comprised 11,898 entries, including flavonoids, steroids, phenylpropanoids, natural-product derivatives and compounds of other structural types. Compounds are prepared under the Prepare Ligands module. In this process, ionization was obtained via generating possible states at target pH: 7.5 ± 2.0. Additionally, conditions of generating tautomers and isomers were defaulted. Compounds were ranked by LibDock score, and the top 96 candidates were further subjected to CDOCKER docking for thorough conformational sampling and pose optimization within the identical binding-site sphere. CDOCKER is a molecular docking method which employs a CHARMm force field. This force field was applied for both receptors and ligands to produce high-precision docking results. The ligand STX-478 exhibited a high conformational consistency between the docking model and the crystal structure, with an RMSD of less than 1.0 Å. The CDOCKER method was adopted to analyze the CDOCKER energy values and binding interactions of the candidate molecules with the protein allosteric pocket. Ultimately, several candidates possessing favorable docking scores were screened out. CDOCKER energy is the total docking energy calculated and its core function is to perform relative ranking for different ligands or conformations during the docking workflow, rather than serving as a rigorous thermodynamic physical quantity.

### 2.3. ADMET Studies

The structural characteristics of drug molecules determine their physicochemical and biochemical properties, ultimately influencing their pharmacokinetic and toxicological profiles. In this section, ADMETlab 3.0 (https://admetmesh.scbdd.com/, accessed on 16 April 2026) and SwissADME (http://www.swissadme.ch/, accessed on 25 April 2026) were employed to evaluate Lipinski’s Rule of Five parameters and pharmacokinetic properties of the molecules. The assessment included MW, LogP, nRot, nHA, nHD, and toxicity profiles (hepatotoxicity, neurotoxicity, etc.). In silico toxicity assessment of rosmarinic acid was performed via the ProTox-3.0 (https://tox.charite.de/protox3/, accessed on 16 April 2026) and ADMETlab 3.0 databases (accessed on 16 April 2026) [41]. The physicochemical properties of the candidate compounds were further verified using ADMETlab 3.0.

### 2.4. Molecular Dynamics Simulation

Molecular dynamics simulations were carried out with GROMACS 2020.3. The amber99sb-ildn force field was applied, while the GAFF force field was adopted to generate topological parameters. For two independent replicates, independently assigned initial velocities were generated randomly, with random seeds applied. The ligand parametrization procedure was performed using the RESP charge model. The ionic concentration was maintained at 0.15 M. The simulation box had dimensions of 13 nm × 13 nm × 13 nm, with an atom count of 216,089 for system. A cutoff of 1.2 nm was used for non-bonded interactions. A simulation box was constructed with a minimum distance of over 1 nm between any protein atom and the box boundary, which was solvated with SPC216 water molecules and neutralized by adding Na^+^ and Cl^−^ ions. The system was first minimized via steepest descent optimization to eliminate atomic clashes. To achieve sufficient pre-equilibration of the simulation system, the NVT ensemble and the NPT ensemble were performed for 100 ps at 300 K and 1 bar, respectively. The MD simulation of 100 ns was performed with periodic boundary conditions, and the temperature (300 K) and pressure (1 bar) were controlled by the V-rescale and Parrinello–Rahman methods, respectively. The leapfrog algorithm with a 2 fs timestep was employed, PME was used for long-range electrostatic calculations, and LINCS constrained all covalent bonds.

The binding free energy was computed via gmx_mmpbsa [29]. For MMPBSA calculation, frames were extracted every 1 ns from the last 25 ns segment of each converged trajectory. The solvent dielectric constant (sdie) was set to 78.54 and the solute dielectric constant (pdie) was set to 2. The ionic strength was 0.15 M, with cation radius of 0.95 Å and anion radius of 1.81 Å. The interaction-entropy scheme was utilized for entropy calculation. Each independent replicate was analyzed separately. Mean values together with standard deviation across the two replicates were reported.

### 2.5. Analysis of Metabolic Pathways and Metabolites

GLORYx (https://nerdd.univie.ac.at/gloryx, accessed on 1 May 2026) was adopted to predict its metabolic pathways and downstream metabolites [42]. The covered metabolic processes mainly contained phase I and phase II metabolic reactions. After uploading the SDF structural file of compounds, relevant metabolic analysis was subsequently carried out.

## 3. Results

### 3.1. Correlation Analysis Between PIK3CA and Tumors

This study systematically analyzed the gene profiles associated with endometrial cancer, colorectal cancer, HNSCC, and breast cancer using the GeneCards database. The search results revealed that the number of genes linked to endometrial cancer, colorectal cancer, HNSCC, and breast cancer were 2164, 11,471, 2161, and 50,000, respectively, with relevance scores ranging from 0.20–88.65, 0.17–193.88, 0.13–12.88, and 0.51–313.78, respectively. The relevance score is positively correlated with the strength of gene–disease associations. Notably, the *PIK3CA* gene exhibited remarkable clinical relevance, with relevance scores of 20.91 (endometrial cancer), 50.87 (colorectal cancer), 9.23 (HNSCC), and 73.08 (breast cancer) (Supplementary Data S1–S4).

### 3.2. Virtual Screening

In this section, we first constructed a PI3Kα allosteric inhibitor screening model to screen a compound library containing 11,898 small molecules. Rapid virtual screening was performed using the LibDock module, and the top 96 compounds (LibDock score ≥ 144.486) were retained after screening (Table S1). Subsequently, the 96 compounds were re-docked using the CDOCKER docking method. In molecular docking research, CDOCKER energy is an important scoring criterion that comprehensively considers the strain energy inside the molecule and the interaction energy between the receptor and ligand. Therefore, a lower CDOCKER energy typically indicates more stable binding modes and stronger interactions. To verify the reliability of docking protocol and confirm the spatial overlap between docked conformations and the crystallographic binding pose, we calculated the heavy-atom RMSD between the docked STX-478 and the co-crystallized STX-478 within PI3Kα allosteric site. The RMSD was calculated as 0.3340 Å, indicating that the docking protocol is capable of reproducing the binding pose of the co-crystallized ligand (Figure S1). We selected the allosteric inhibitor STX-478 as the positive control. Multiple compounds including rosmarinic acid (compound 5281792), curcumin (compound 969516), pentanochelin (compound 15774019), vermiculinic acid (compound 6440374), uridine 5′-triphosphate (compound 6133), and others were found to bind to the PI3Kα allosteric pocket. Rosmarinic acid exhibits a lower CDOCKER energy than STX-478 (Table 1).

**Figure 3:**
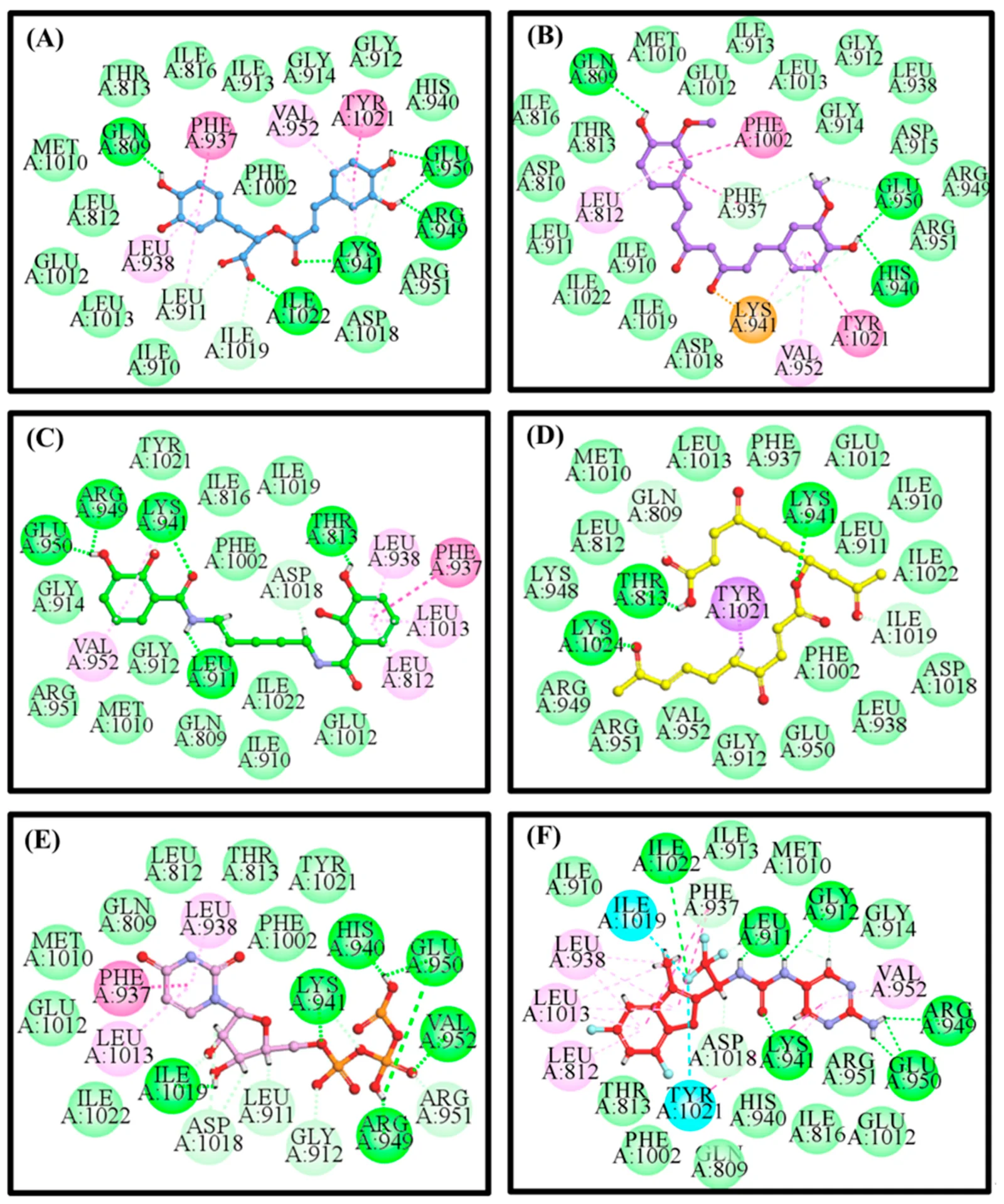
The interaction diagrams of rosmarinic acid (**A**), curcumin (**B**), pentanochelin (**C**), vermiculinic acid (**D**), uridine 5′-triphosphate (**E**) and STX-478 (**F**) bound to PI3Kα. Dark green dashed line: conventional hydrogen bond; light green dashed line: carbon hydrogen bond; pink/pale purple/purple dashed line: pi interactions; orange dashed line: salt bridge.

Figure 3 showed the binding patterns of rosmarinic acid, curcumin, pentanochelin, vermiculinic acid, uridine 5′-triphosphate, and STX-478 to allosteric sites. Rosmarinic acid (Figure 3A), curcumin (Figure 3B), pentanochelin (Figure 3C), vermiculinic acid (Figure 3D), uridine 5′-triphosphate (Figure 3E), and STX-478 (Figure 3F) could all form hydrogen bonds with LYS941. In addition, rosmarinic acid formed hydrogen bonds with GLN809, LEU911, ARG949, GLU950, ILE1019 and ILE1022; curcumin interacted with GLN809, PHE937, HIS940, and GLU950 through hydrogen bonds; pentanochelin bound THR813, LEU911, ARG949, GLU950, and ASP1018 via hydrogen bonding; vermiculinic acid created hydrogen bond interactions with GLN809, THR813, ILE1019 and LYS1024, while uridine 5′-triphosphate formed hydrogen bonds with LEU911, GLY912, HIS940, ARG949, GLU950, VAL952, ASP1018, and ILE1019.

**Figure 4:**
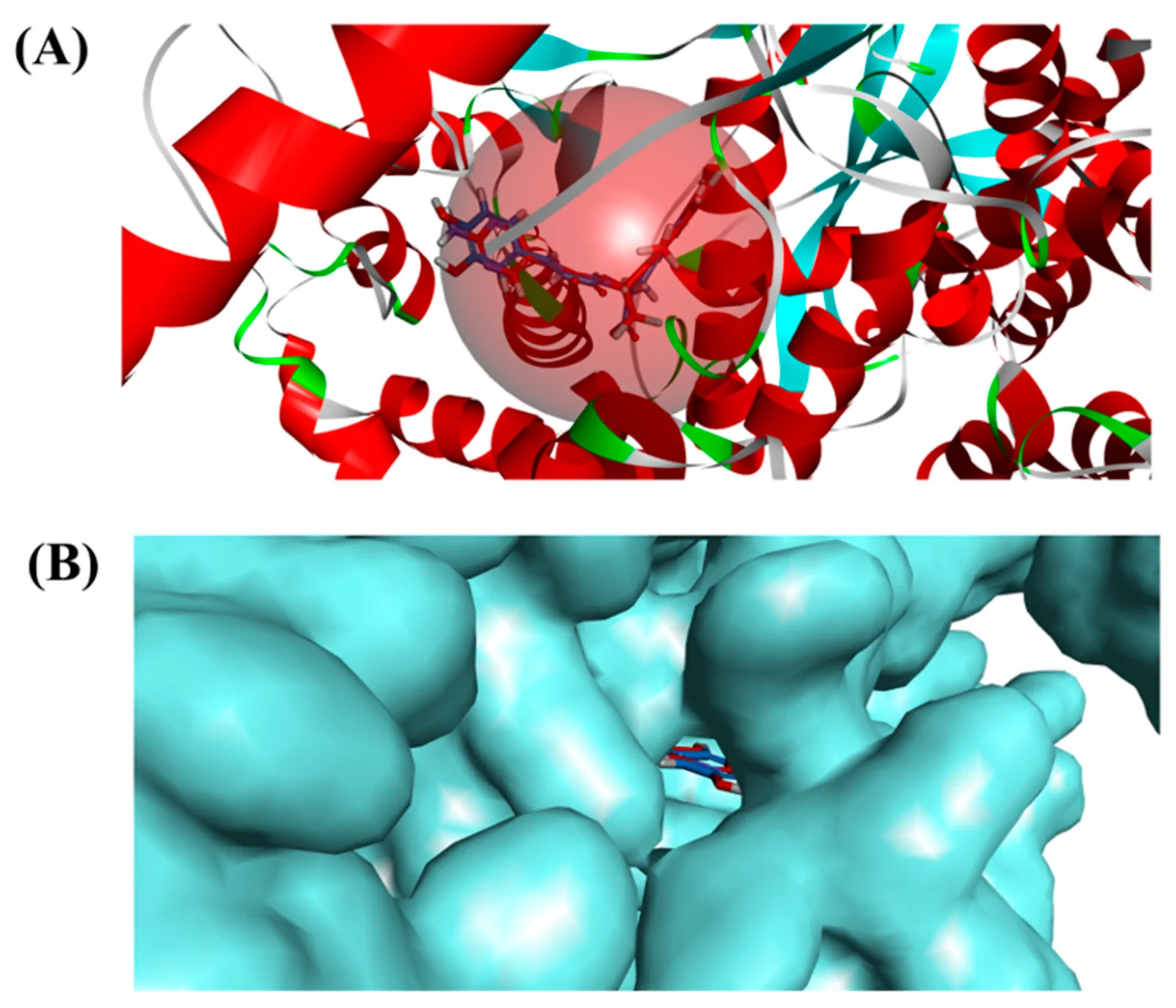
Overlay diagram of rosmarinic acid, STX-478 with the allosteric site of PI3Kα: solid ribbon representation (**A**) and surface rendering representation (**B**). The red spheres indicate the binding site.

Rosmarinic acid exhibited the best docking score for PI3Kα. In the rosmarinic acid structure, the hydrogen atom of the hydroxyl group forms a hydrogen bond with the backbone carbonyl group of GLN809, with a hydrogen-bond distance of 2.7 Å. The oxygen atom of the carboxyl group forms hydrogen bonds with backbone hydrogen atoms of LEU911, ILE1019, and ILE1022, with distances of 2.6 Å, 2.8 Å, and 2.5 Å, respectively. In addition, the backbone hydrogen atom of LYS941 can form a hydrogen bond with the oxygen atom of the hydroxyl group, with a distance of 2.53 Å. The backbone carbonyl of Glu950 form hydrogen bonds with the hydrogen atom of the phenolic hydroxyl group, with distances of 2.4 Å. The backbone carbonyl of ARG949 also interacts with the hydrogen atom of the phenolic hydroxyl group via a hydrogen-bond interaction at 2.6 Å. Analogous to STX-478, it could bind to allosteric sites, and also stably bound residues LEU911 and LYS941 (Figure 3 and Figure 4), indicating promising potential for subsequent research.

### 3.3. The Evaluation of Druggability

**Figure 5:**
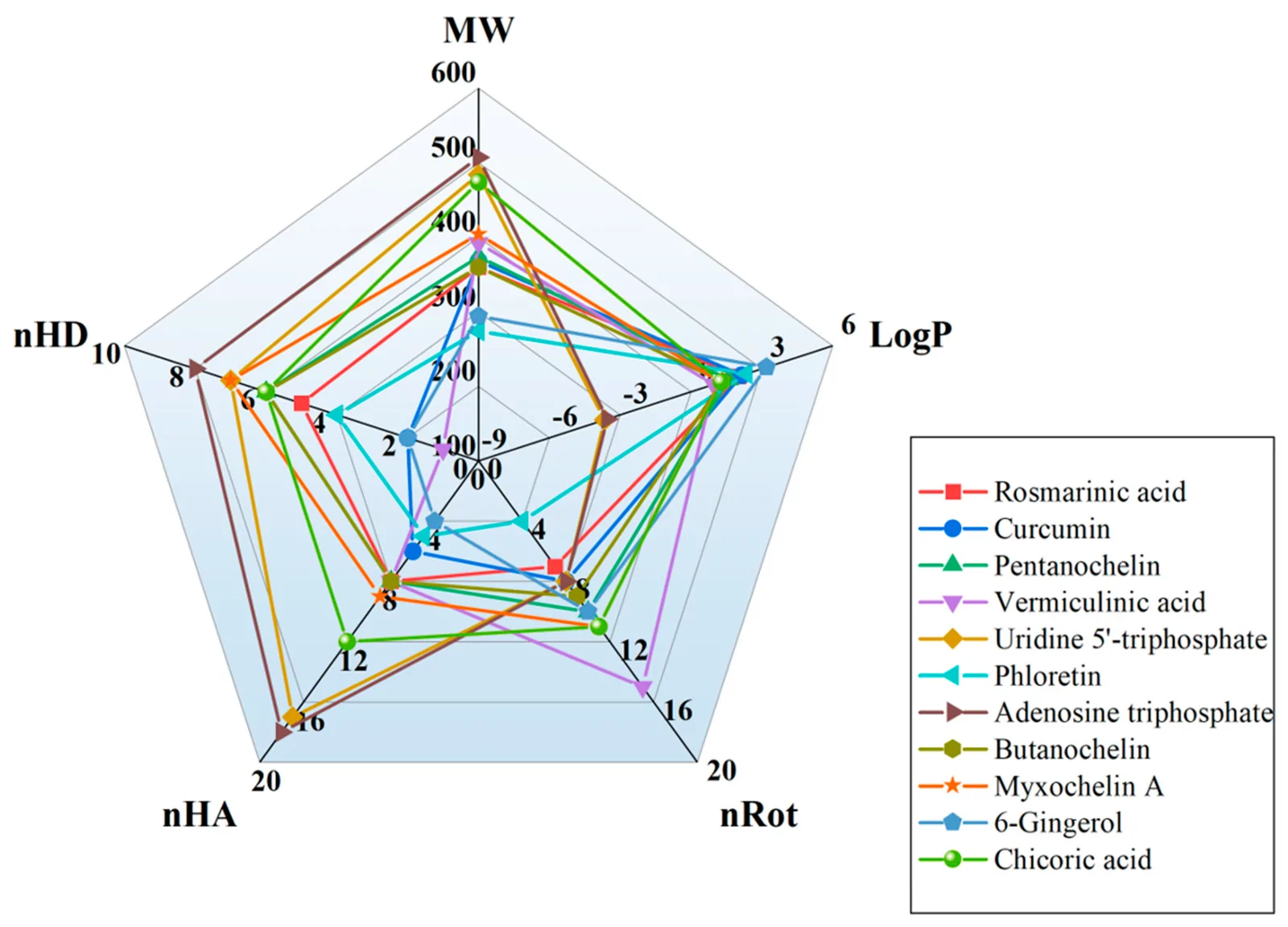
The Lipinski’s Rule of Five of screened compounds: 1. MW ≤ 500 Da; 2. LogP ≤ 5; 3. nRot: 0~11; 4. nHA: 0~10; 5. nHD: 0~5.

Lipinski’s Rule of Five is an important empirical rule in the field of medicinal chemistry, proposed by Pfizer scientist Christopher Lipinski in 1997. It is used to evaluate the potential of small molecule compounds to become oral medications. As illustrated in Figure 5 and Table S2, the compounds that do not violate Lipinski’s rule of five are rosmarinic acid, curcumin, phloretin and 6-gingerol.

Next, we conducted a detailed evaluation of the ADMET properties of rosmarinic acid. ADMET properties were predicted using ADMETlab 3.0 and SwissADME to preliminarily screen compounds with favorable drug likeness. Plasma protein binding (PPB) is one of the key parameters affecting the pharmacokinetic properties of drugs. The interaction between drug molecules and plasma proteins exerts a significant impact on their pharmacological efficacy, as only the free-form drugs can exert therapeutic effects and participate in metabolic processes. PPB > 90% represents strong binding ability [43]. The PPB of rosmarinic acid was predicted to be 77.3%, indicating that it may exhibit moderate plasma protein binding. Plasma clearance (CL), a key pharmacokinetic parameter, plays a vital role in assessing the efficiency of drug metabolism and excretion processes. A CL value below 5 mL/min/kg suggests that the drug is cleared from the body at a low rate, which may lead to an extended half-life and potential accumulation risks. The predicted CL values of rosmarinic acid was 13.238 mL/min/kg, indicating that rosmarinic acid may be a moderate-clearance drug (Table 2). The plasma elimination half-life (T_1/2_) is an important pharmacokinetic parameter reflecting the drug metabolism and clearance rate. The predicted T_1/2_ values of rosmarinic acid is 1.906 h, indicating that compound may be a short-half-life drug (Table 2). The predicted bioavailability score and Log *K*p of rosmarinic acid were 0.56 and −6.82 cm/s, respectively (Table 2). Prediction results suggested that rosmarinic acid has no inhibitory effects on cytochrome P450 (CYP450) enzymes (CYP3A4, CYP1A2, etc.) (Table 2). The possibility of rosmarinic acid acting as a Pgp inhibitor was also relatively low (Table 2).

**Figure 6:**
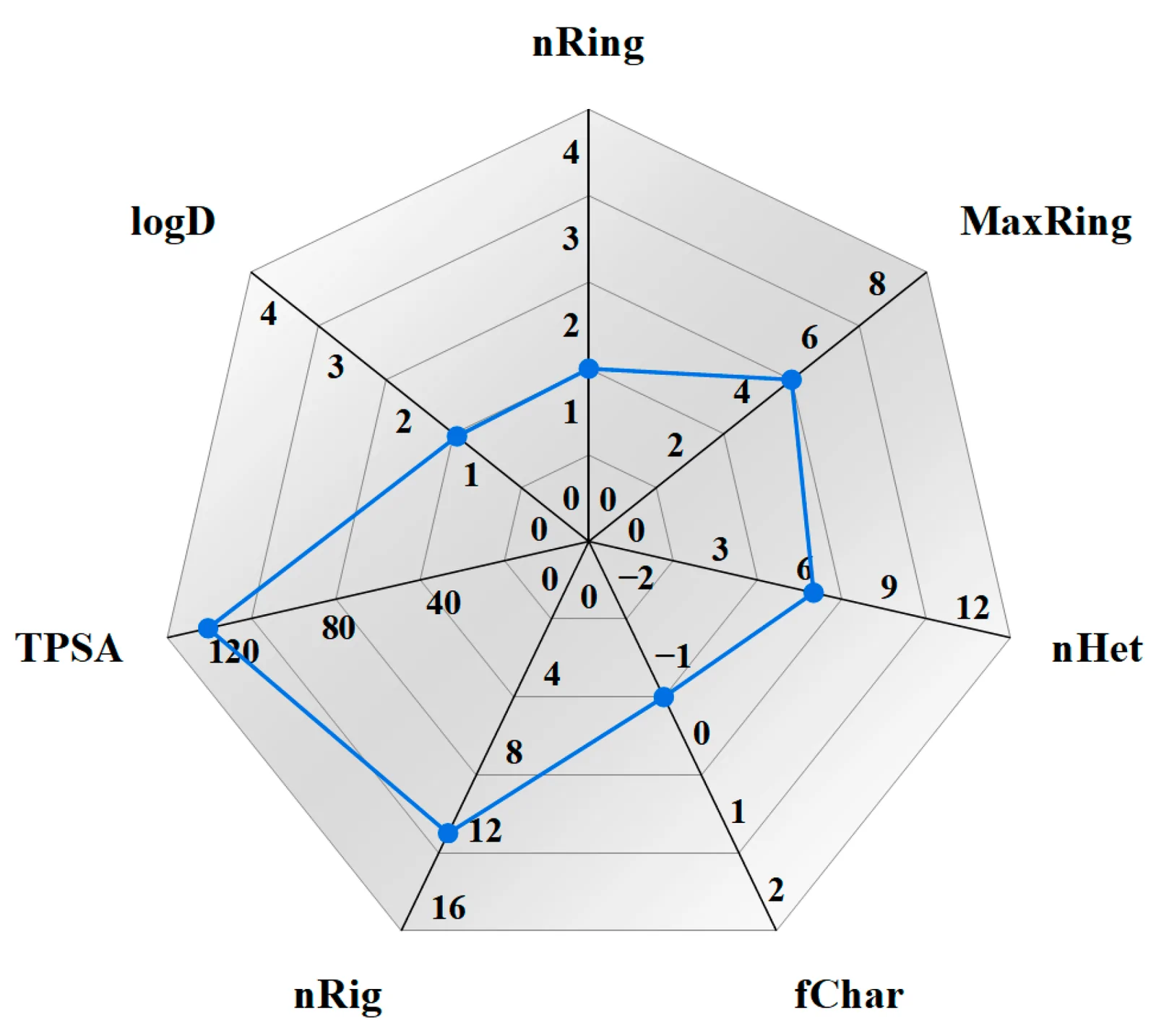
The physicochemical properties prediction of rosmarinic acid.

An LD_50_ value > 2000 mg/kg signifies low toxicity, whereas an LD_50_ < 500 mg/kg indicates significant toxicity. The predicted LD_50_ of rosmarinic acid was 5000 mg/kg, implying that it might exhibit relatively low toxicity (Table 3). In the toxicity assessment, it was found that the probabilities of rosmarinic acid exhibiting no hepatotoxicity, neurotoxicity, respiratory toxicity, cardiotoxicity, carcinogenicity, mutagenicity, and cytotoxicity were 62%, 88%, 54%, 69%, 66%, 85%, and 90%, respectively (Table 3). In addition, rosmarinic acid exhibited lower probabilities of causing cardiotoxicity (hERG blocker), carcinogenicity, eye corrosion, neurotoxicity, and hematotoxicity (Table S3). The drug-like properties were further evaluated by ADMETlab 3.0. Results indicated that rosmarinic acid exhibited qualified physicochemical profiles for good druggability (Figure 6).

### 3.4. Molecular Dynamics Simulation of PI3Kα/Rosmarinic Acid-PI3Kα System

**Figure 7:**
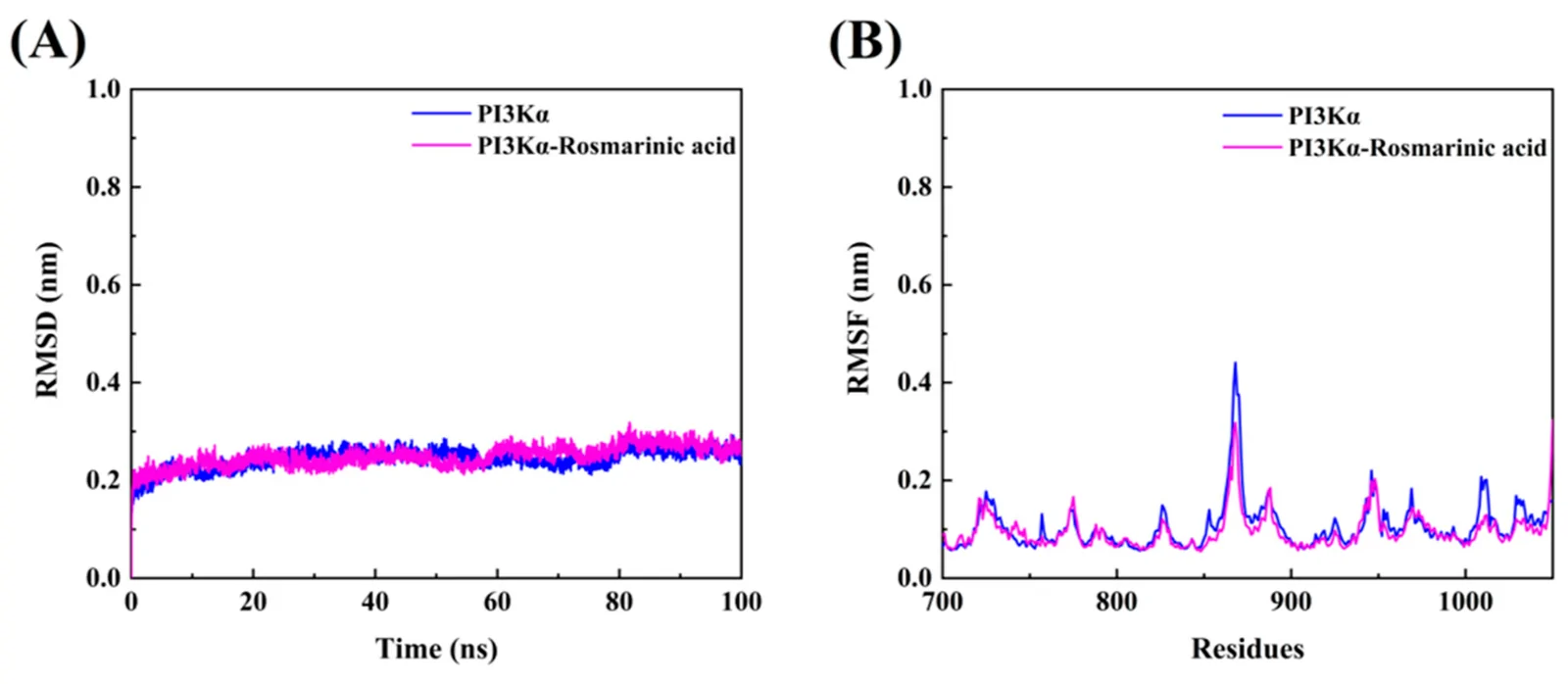
(**A**) RMSD profiles of PI3Kα/PI3Kα–rosmarinic acid from one independent MD replicate. (**B**) RMSF profiles of PI3Kα/PI3Kα–rosmarinic acid from one independent MD replicate.

Root mean square deviation (RMSD) serves as a critical indicator of structural stability in molecular dynamics simulations, measuring how the coordinates of selected atoms fluctuate relative to a reference conformation. A plateau in RMSD values typically signifies that the simulated system has reached a stable equilibrium state [30]. As shown in Figure 7A and Figure S2A, both PI3Kα and the PI3Kα–rosmarinic acid complex reached equilibrium after 20 ns, with RMSD values of 0.247 ± 0.005 nm and 0.255 ± 0.001 nm, respectively. These results indicate that both simulation systems achieved stable conformational equilibrium. Root mean square fluctuation (RMSF) is used to evaluate the positional fluctuation of each atom around its average location [44,45,46]. Figure 7B and Figure S2B and Table S4 show that the RMSF values of PI3Kα and the PI3Kα–rosmarinic acid complex were 0.119 ± 0.008 nm and 0.108 ± 0.010 nm, respectively.

**Figure 8:**
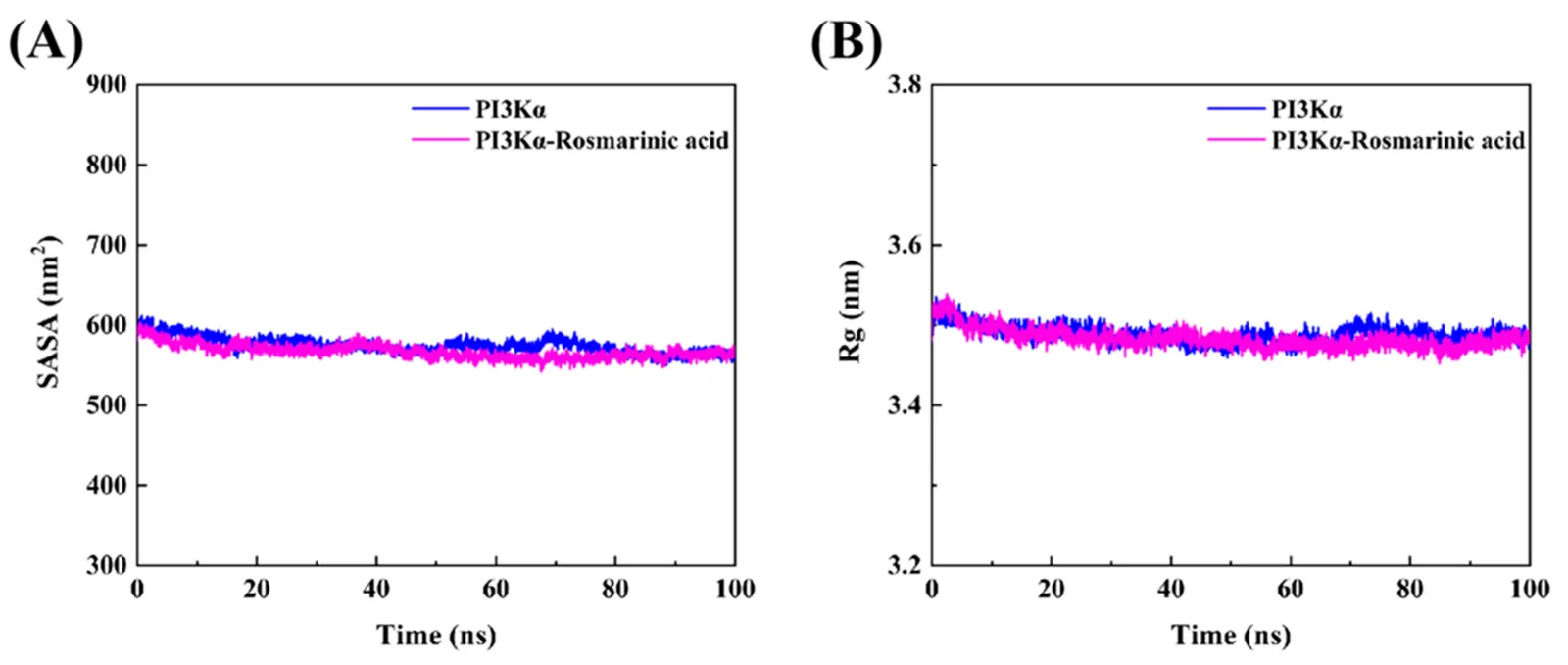
(**A**) The SASA of PI3Kα/PI3Kα–rosmarinic acid from one independent MD replicate. (**B**) The Rg of PI3Kα/PI3Kα–rosmarinic acid from one independent MD replicate.

As shown in Figure 8A and Figure S3A and Table S4, the SASA value of PI3Kα in PI3Kα–rosmarinic acid showed a downward trend during the simulation. The average SASA values of the PI3Kα and PI3Kα–rosmarinic acid systems were 573.514 ± 2.650 nm^2^ and 570.475 ± 8.554 nm^2^, respectively. Figure 8B and Figure S3B and Table S4 show the radius of gyration (Rg) values of PI3Kα/PI3Kα–rosmarinic acid. The average Rg values of the PI3Kα and PI3Kα–rosmarinic acid systems were 3.485 ± 0.001 nm and 3.478 ± 0.002 nm, respectively. Lower RMSF values were observed for the PI3Kα–rosmarinic acid complex, suggesting that rosmarinic-acid binding may enhance the structural stability of PI3Kα. Consistently, slight decreases in SASA and Rg further supported a more compact protein conformation upon ligand binding.

We adopted the MMPBSA approach to dissect the binding energy of PI3Kα–rosmarinic acid complexes. The total binding energy was partitioned into electrostatic, van der Waals, polar solvation and nonpolar solvation terms, and all data are listed in Table S5. The results revealed a binding free energy of −29.763 ± 5.691 kJ/mol for the PI3Kα–rosmarinic acid system. The negative binding free-energy indicated that formation of the PI3Kα–rosmarinic acid complex was thermodynamically favorable under simulation conditions.

**Figure 9:**
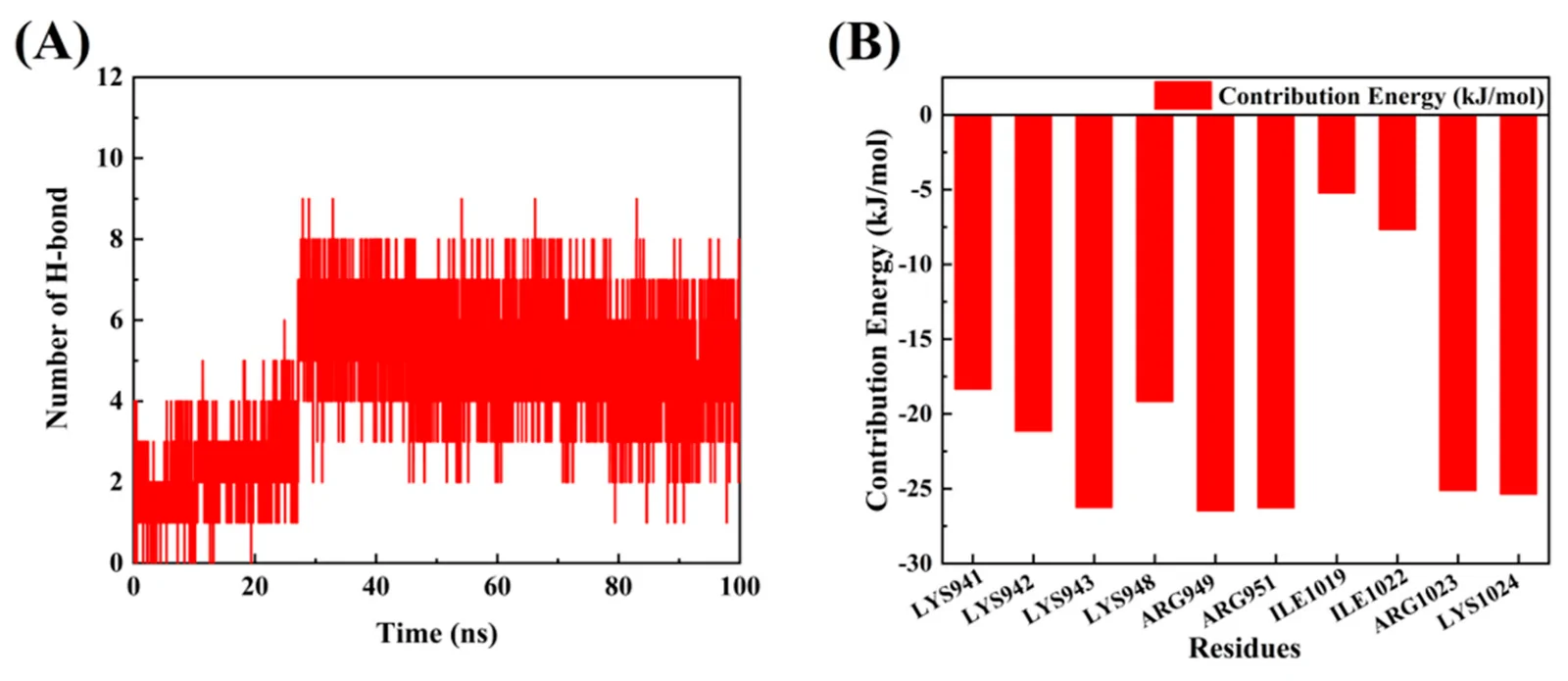
(**A**) The curve of the number of H-bonds of PI3Kα–rosmarinic acid from one independent MD replicate. (**B**) Residual contribution energy of the interaction between PI3Kα and rosmarinic acid from one independent MD replicate.

We further analyzed hydrogen bond interactions to probe the binding mode of PI3Kα with rosmarinic acid after simulation equilibration. The mean hydrogen bond counts was 3.971 ± 1.612, verifying stable hydrogen-bonding contacts between the PI3Kα and rosmarinic acid (Figure 9A and Figure S4A and Table S4). We also analyzed the per-residue energy contribution to clarify detailed interactions between rosmarinic acid and the allosteric pocket of PI3Kα. According to Figure 9B, Figures S4B and S5, residues LYS941, LYS942, LYS943, LYS948, ARG949, ARG951, ILE1019, ILE1022, ARG1023, and LYS1024 constitute the core interacting residues of the PI3Kα–rosmarinic acid complex.

### 3.5. Predicted Metabolic Profile of Rosmarinic Acid

**Figure 10:**
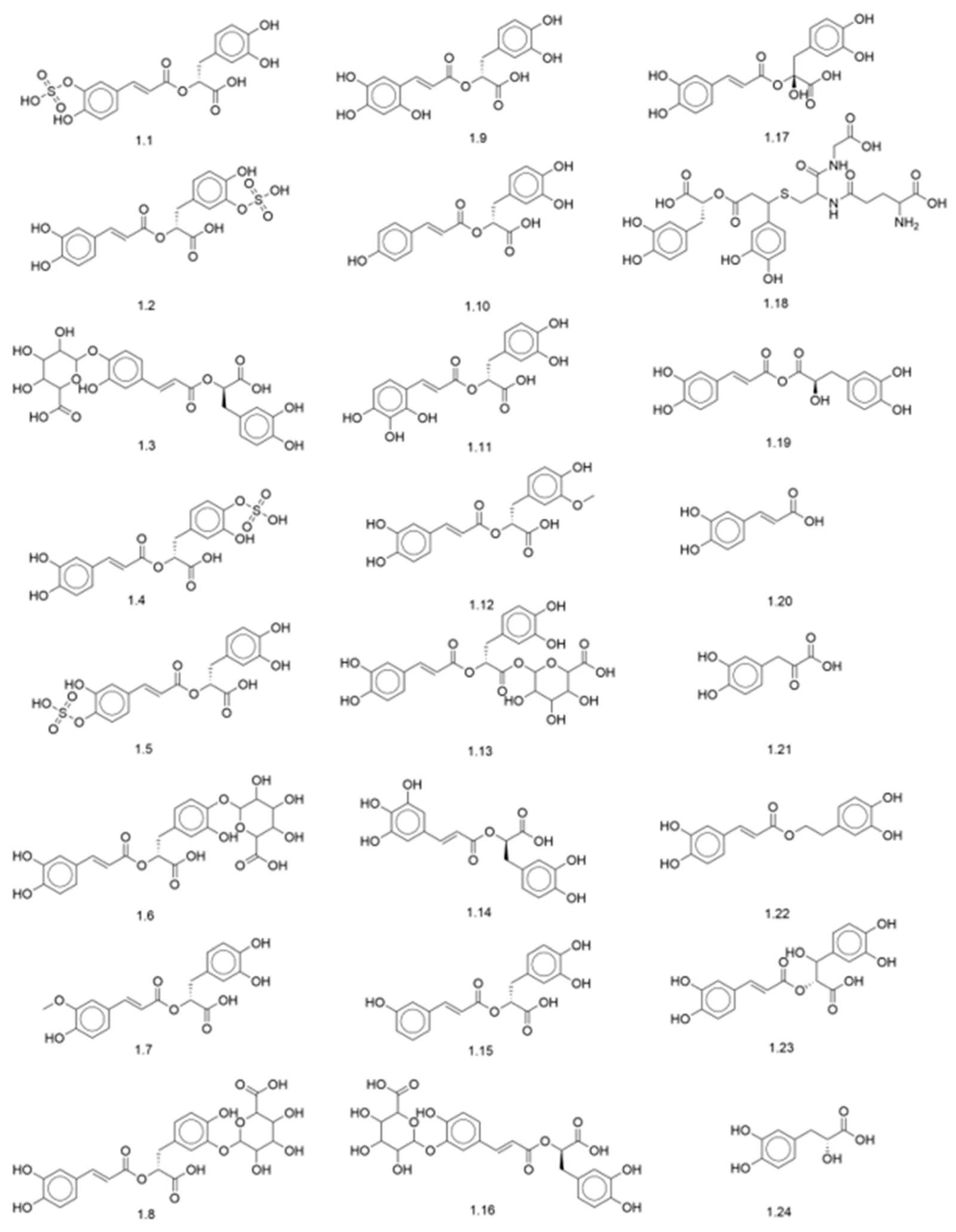
Predicted metabolites (**1.1**–**1.24**) of rosmarinic acid.

**Figure 11:**
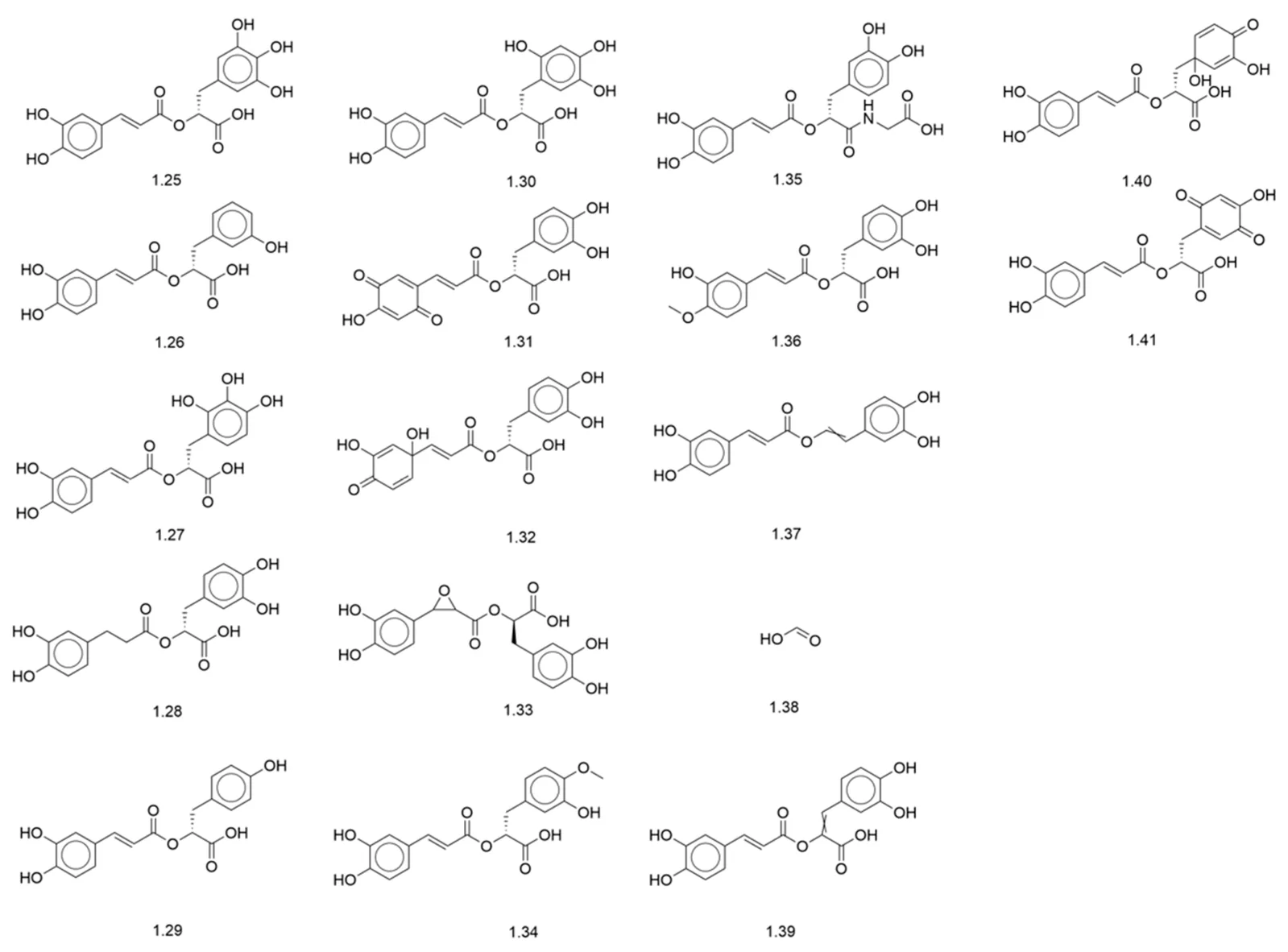
Predicted metabolites (**1.25**–**1.41**) of rosmarinic acid.

The predicted metabolic profiles showed that rosmarinic acid might undergo phase I/II metabolic transformations in the body, covering key pathways such as sulfation, O-glucuronidation, methylation, aromatic hydroxylation, aromatic dehydroxylation, and so on (Figure 10 and Figure 11). Rosmarinic acid is a natural phenolic acid compound. Its structure contains a carboxylic acid group and two catechol structural units, which are the main sites for metabolic reactions. In total, 41 metabolites are generated through the above metabolic pathways, among which 6 metabolites have priority score (Supplementary Data S5) greater than 0.8, namely (*R*,*E*)-3-(3,4-dihydroxyphenyl)-2-((3-(4-hydroxy-3-(sulfooxy)phenyl)acryloyl)oxy)propanoic acid (**1.1**), (*R*,*E*)-2-((3-(3,4-dihydroxyphenyl)acryloyl)oxy)-3-(4-hydroxy-3-(sulfooxy)phenyl)propanoic acid (**1.2**), 6-(4-((*E*)-3-((R)-1-carboxy-2-(3,4-dihydroxyphenyl)ethoxy)-3-oxoprop-1-en-1-yl)-2-hydroxyphenoxy)-3,4,5-trihydroxytetrahydro-2H-pyran-2-carboxylic acid (**1.3**), (*R*,*E*)-2-((3-(3,4-dihydroxyphenyl)acryloyl)oxy)-3-(3-hydroxy-4-(sulfooxy)phenyl)propanoic acid (**1.4**), (*R*,*E*)-3-(3,4-dihydroxyphenyl)-2-((3-(3-hydroxy-4-(sulfooxy)phenyl)acryloyl)oxy)propanoic acid (**1.5**), and 6-(4-((*R*)-2-carboxy-2-(((*E*)-3-(3,4-dihydroxyphenyl)acryloyl)oxy)ethyl)-2-hydroxyphenoxy)-3,4,5-trihydroxytetrahydro-2H-pyran-2-carboxylic acid (**1.6**). The priority scores are 0.948, 0.944, 0.94, 0.936, 0.872, and 0.816, respectively. These 6 metabolites are mainly produced through the sulfation and O-glucuronidation pathways.

## 4. Conclusions

This study focuses on the development needs of novel PI3Kα allosteric inhibitors, with a focus on optimizing drug selectivity and safety. A computer-aided screening-based PI3Kα allosteric inhibitor discovery platform has been established. By integrating molecular docking, multidimensional ADMET prediction, and molecular dynamics simulation techniques, a small molecule library was systematically screened, and predicted ligands of the PI3Kα allosteric pocket were obtained. Rosmarinic acid is a natural phenolic acid compound, in which the hydroxyl group on the benzene ring can form hydrogen bonds with GLN809, GLU950, ARG949, and LYS941 in the allosteric site, the carbonyl group can form hydrogen bond with LYS941, and the carboxyl group can form hydrogen bonds with ILE1022, ILE1019, and LEU911. Rosmarinic acid possesses favorable safety characteristics and acceptable predicted pharmacokinetic properties.

Rosmarinic acid undergoes extensive phase II conjugation dominated by sulfation and O-glucuronidation, and this process likely lowers systemic exposure of the parent compound. Multiple ionizable phenolic hydroxyl groups and a carboxylic acid reduce membrane permeability, further limiting oral absorption. Since sulfation/glucuronidation greatly alter molecular properties, it is speculated that conjugated metabolites may lose their strong PI3Kα binding affinity. Collectively, rosmarinic acid is better considered an attractive starting scaffold, and structural modification may be needed to overcome poor permeability and extensive metabolism.

This study is restricted to computational prediction and lacks supporting biological experimental validation. Notably, the kinase inhibitory potency, isoform selectivity, and cellular activity of rosmarinic acid remain uncharacterized. Comprehensive preliminary in vitro characterizations are warranted, including testing inhibition against recombinant wild-type and H1047R mutant PI3Kα, generating concentration–response curves to determine IC_50_ values, conducting counter-screening against PI3Kβ, PI3Kδ and PI3Kγ, measuring p-AKT suppression in *PIK3CA*-mutant cell lines with parallel comparisons in *PIK3CA*-wild-type cell lines, and excluding non-specific aggregation or assay interference commonly associated with polyphenolic compounds. Follow-up in vitro and in vivo experiments will be implemented to verify whether rosmarinic acid exerts anti-tumor activity via specific binding to the allosteric pocket of PI3Kα. Moreover, X-ray cocrystal structure determination of PI3Kα–rosmarinic acid complex should be pursued to experimentally validate the computationally predicted allosteric binding pose and intermolecular interactions within the allosteric pocket. Collectively, the present work provides a computational basis for discovering candidate allosteric PI3Kα inhibitors.

## Figures and Tables

**Table 1:** Compound structures and docking results.

Name	Chemical Structure	Molecular Formula	CDOCKER Energy (kcal/mol)
Rosmarinic acid (Compound 5281792)	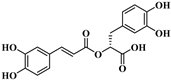	C_18_H_16_O_8_	−69.4529
Curcumin (Compound 969516)	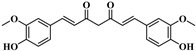	C_21_H_20_O_6_	−63.9918
Pentanochelin (Compound 15774019)	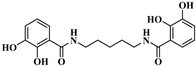	C_19_H_22_N_2_O_6_	−57.524
Vermiculinic acid (Compound 6440374)	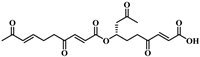	C_20_H_24_O_8_	−56.2194
Uridine 5′-triphosphate (Compound 6133)	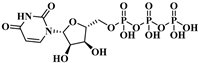	C_9_H_15_N_2_O_15_P_3_	−56.1629
Phloretin (Compound 4788)	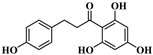	C_15_H_14_O_5_	−54.9223
Adenosine triphosphate (Compound 5957)	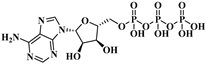	C_10_H_16_N_5_O_13_P_3_	−54.8551
Butanochelin (Compound 10338559)	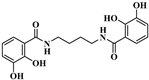	C_18_H_20_N_2_O_6_	−54.8389
Myxochelin A (Compound 16093504)	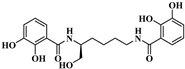	C_20_H_24_N_2_O_7_	−54.5735
6-Gingerol (Compund 442793)	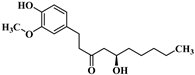	C_17_H_26_O_4_	−53.5644
Chicoric acid (Compund 5281764)	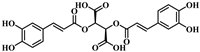	C_22_H_18_O_12_	−51.0527
STX-478	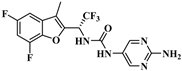	C_16_H_12_F_5_N_5_O_2_	−66.7128

**Table 2:** The ADME properties prediction of the rosmarinic acid.

	Rosmarinic Acid
PPB ^1^ (%)	77.3%
BBB ^2^ (%)	No
CL_plasma_ ^3^ (mL/min/kg)	13.238
T_1/2_ ^4^ (h)	1.906
Bioavailability score ^5^	0.56
Log Kp ^6^ (cm/s)	−6.82
VDss ^7^ (L/kg)	0.451
CYP2D6 inhibitor ^8^	No
CYP1A2 inhibitor ^8^	No
CYP2C19 inhibitor ^8^	No
CYP2C9 inhibitor ^8^	No
CYP3A4 inhibitor ^8^	No
Pgp inhibitor ^9^ (%)	0–10%

1. PPB: optimal: <90%. 2. BBB: blood–brain barrier penetration. 3. CL_plasma_: >15 mL/min/kg: high clearance; 5–15 mL/min/kg: moderate clearance; <5 mL/min/kg: low clearance. 4. T_1/2_: ultra-short-half-life drugs: T_1/2_ < 1 h; short-half-life drugs: T_l/2_ between 1–4 h; intermediate short-half-life drugs: T_l/2_ between 4–8 h; long-half-life drugs: T_l/2_ > 8 h. 5. Bioavailability score: probability of F > 10% in rat. 6. Log Kp: skin permeation value. 7. VDss: volume distribution; optimal: 0.04–20 L/kg. 8. CYP2D6 inhibitor, CYP1A2 inhibitor, CYP2C19 inhibitor, CYP2C9 inhibitor and CYP3A4 inhibitor: ‘no’ represents no inhibitory effect on the enzyme and ‘Yes’ indicates an inhibitory effect on the enzyme. 9. Pgp inhibitor: the probability of being Pgp inhibitor.

**Table 3:** ProTox-3.0-based toxicity prediction of rosmarinic acid.

	Rosmarinic Acid
LD_50_ (mg/kg)	5000
Hepatotoxicity	Inact62%
Neurotoxicity	Inact88%
Respiratory toxicity	Inact54%
Cardiotoxicity	Inact69%
Carcinogenicity	Inact66%
Mutagenicity	Inact85%
Cytotoxicity	Inact90%

Inact%: Non-toxic prediction rate of the compound.

## Data Availability

Data is provided within the manuscript or Supplementary Materials.

## Supplementary Materials

The following supporting information can be downloaded at: https://www.mdpi.com/article/10.3390/biology15211935/s1, Figure S1: The docking pose of STX-478 overlaps with the crystallographic ligand pose. The red stick represents the docked structure of STX-478, and the gray stick corresponds to the original STX-478 structure in the protein crystal. Figure S2: (A) RMSD profiles of PI3Kα/PI3Kα-rosmarinic acid from the second independent MD replicate. (B) RMSF profiles of PI3Kα/PI3Kα-rosmarinic acid from the second independent MD replicate. Figure S3: (A) The SASA of PI3Kα/PI3Kα-rosmarinic acid from the second independent MD replicate. (B) The Rg of PI3Kα/PI3Kα-rosmarinic acid from the second independent MD replicate. Figure S4: (A) The curve of the number of H-bonds of PI3Kα-rosmarinic acid from the second independent MD replicate. (B) Residual contribution energy of the interaction between PI3Kα and rosmarinic acid from the second independent MD replicate. Figure S5: Representative MD structures of the PI3Kα-rosmarinic acid complex obtained from MD simulation. Table S1: Compounds with higher LibDock Score. Table S2: The evaluation of Lipinski’s Rule of Five for 11 compounds. Table S3: The toxicity assessment results of the rosmarinic acid by ADMETLab 3.0. Table S4: Physical properties calculated from two independent MD replicates. Table S5: The binding free energy (kJ/mol) of PI3Kα with rosmarinic acid, and its components between receptor and ligand. Supplementary Data S1–S4: The genes linked to endometrial cancer (S1), colorectal cancer (S2), HNSCC (S3), and breast cancer (S4). Supplementary Data S5: The metabolic profile of Rosmarinic Acid. Supplementary Data S6: The top 30 compounds ranked by LibDock score.
