# Supplementary material for: In Silico Identification of Rosmarinic Acid as a Putative Natural Product Binder of the PI3Kα Allosteric Site
Source: Biology (Basel). 2026 Oct 25;15(21):1935. doi: 10.3390/biology15211935 (PMC13761470; doi:10.3390/biology15211935)
Supplement: Supplementary file 1 [file biology-15-01935-s001.zip › Figures S1-S5 and Tables S1-S5.pdf]

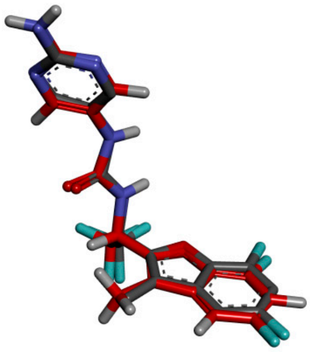

**Figure S1**. The docking pose of STX-478 overlaps with the crystallographic ligand pose. The red stick represents the docked structure of STX-478, and the gray stick corresponds to the original STX-478 structure in the protein crystal.

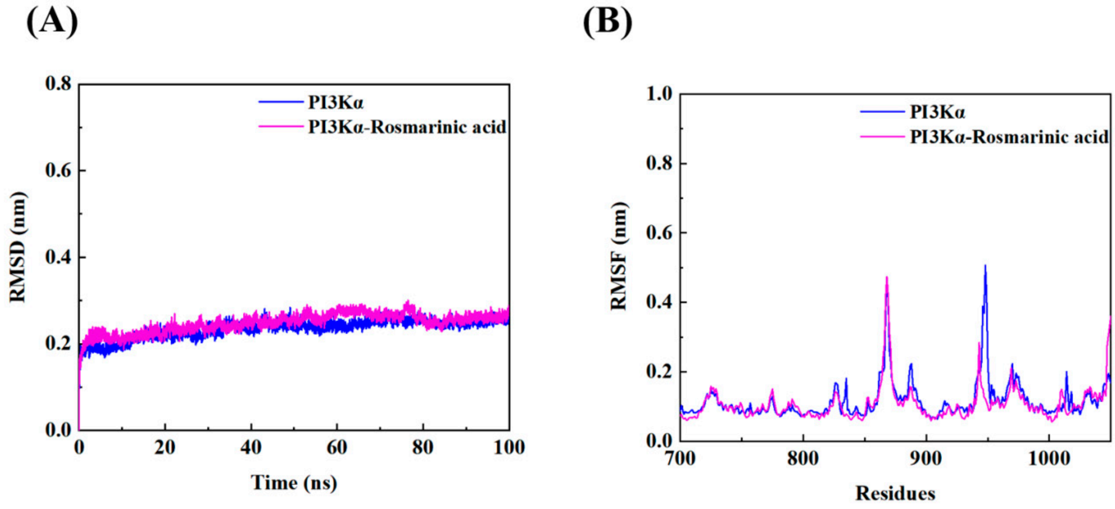


**Figure S2.** (A) RMSD profiles of PI3K$\alpha$/PI3K$\alpha$-rosmarinic acid from the second independent MD replicate. (B) RMSF profiles of PI3K$\alpha$/PI3K$\alpha$-rosmarinic acid from the second independent MD replicate.

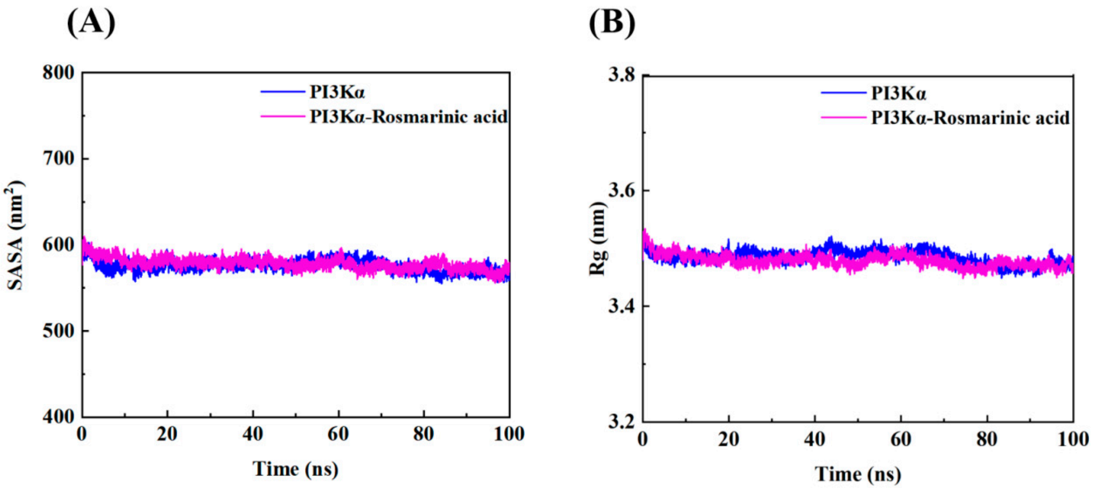


**Figure S3.** (A) The SASA of PI3K$\alpha$/PI3K$\alpha$-rosmarinic acid from the second independent MD replicate. (B) The Rg of PI3K$\alpha$/PI3K$\alpha$-rosmarinic acid from the second independent MD replicate.

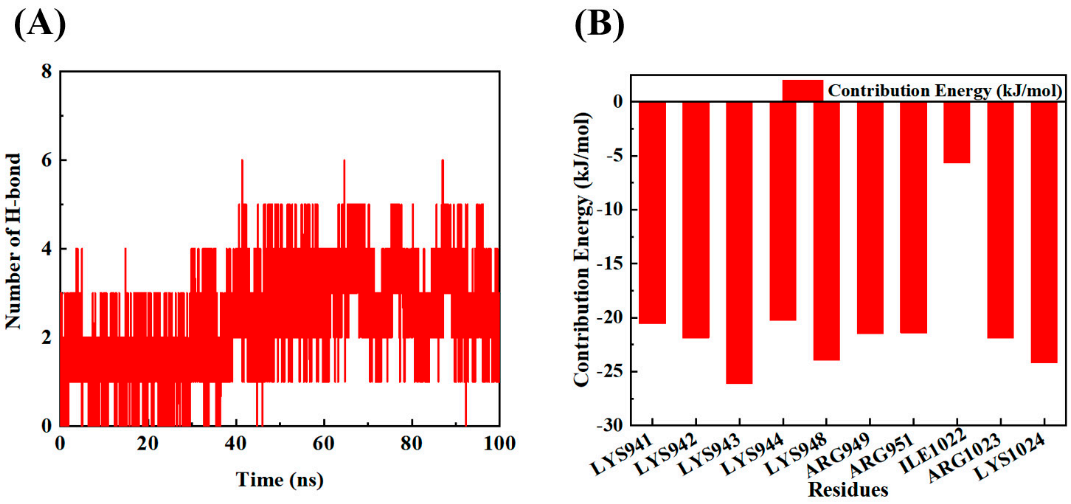


**Figure S4**. (A) The curve of the number of H-bonds of PI3K$\alpha$-rosmarinic acid from the second independent MD replicate. (B) Residual contribution energy of the interaction between PI3K$\alpha$ and rosmarinic acid from the second independent MD replicate.

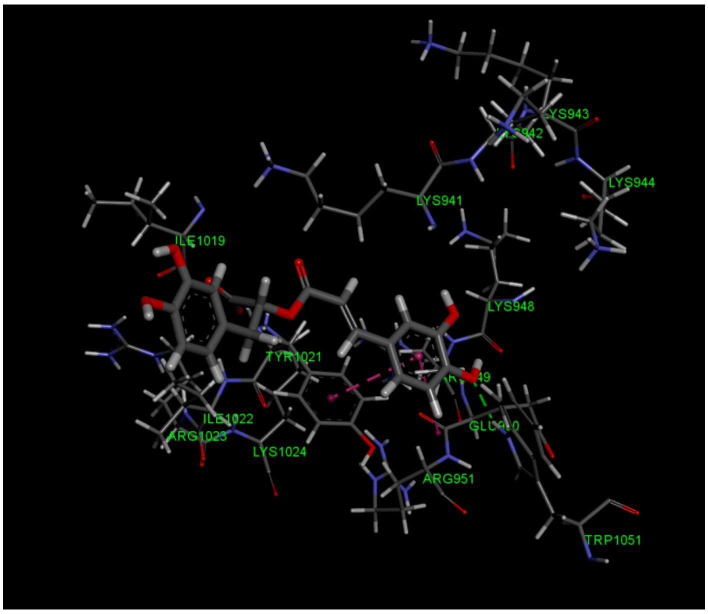


**Figure S5.** Representative MD structures of the PI3Kα-rosmarinic acid complex obtained from MD simulation.

**Table S1**. Compounds with higher LibDock Score.

| Number | Name | LibDock Score |
|---|---|---|
| 1 | Compound 34040 | 171.627 |
| 2 | Compound 5281712 | 171.275 |
| 3 | Compound 65482 | 169.825 |
| 4 | Compound 5957 | 169.492 |
| 5 | Compound 5281764 | 168.43 |
| 6 | Compound 978343 | 167.73 |
| 7 | Compound 122178712 | 164.129 |
| 8 | Compound 129211 | 162.947 |
| 9 | Compound 5281718 | 162.07 |
| 10 | Compound 49831904 | 161.835 |
| 11 | Compound 34756 | 161.816 |
| 12 | Compound 34755 | 161.294 |
| 13 | Compound 5281078 | 161.07 |
| 14 | Compound 637213 | 160.953 |
| 15 | Compound 124072 | 160.092 |
| 16 | Compound 969516 | 159.57 |
| 17 | Compound 6918554 | 159.484 |
| 18 | Compound 5281792 | 159.367 |
| 19 | Compound 7129815 | 158.833 |
| 20 | Compound 6571639 | 158.744 |

| | | |
|---|---|---|
| 21 | Compound 1548994 | 158.057 |
| 22 | Compound 6604001 | 157.977 |
| 23 | Compound 135398658 | 156.564 |
| 24 | Compound 135410875 | 156.455 |
| 25 | Compound 443600 | 156.012 |
| 26 | Compound 4461 | 155.555 |
| 27 | Compound 148177 | 155.237 |
| 28 | Compound 68741582 | 155.166 |
| 29 | Compound 6124753 | 154.52 |
| 30 | Compound 978327 | 154.351 |
| 31 | Compound 10338559 | 153.977 |
| 32 | Compound 16678941 | 153.947 |
| 33 | Compound 4418 | 153.516 |
| 34 | Compound 6957986 | 153.495 |
| 35 | Compound 16086602 | 153.212 |
| 36 | Compound 122178777 | 153.115 |
| 37 | Compound 49865534 | 153.102 |
| 38 | Compound 5280723 | 153.083 |
| 39 | Compound 11669698 | 153.079 |
| 40 | Compound 4735 | 152.947 |
| 41 | Compound 16093504 | 152.577 |
| 42 | Compound 73346043 | 152.413 |
| 43 | Compound 5481350 | 152.412 |
| 44 | Compound 102585017 | 152.369 |
| 45 | Compound 2134 | 152.311 |
| 46 | Compound 15774019 | 151.726 |
| 47 | Compound 7272330 | 151.721 |
| 48 | Compound 978331 | 151.698 |
| 49 | Compound 101176 | 151.561 |
| 50 | Compound 6445533 | 151.516 |
| 51 | Compound 5282136 | 151.411 |
| 52 | Compound 4187769 | 151.088 |
| 53 | Compound 2372 | 150.946 |
| 54 | Compound 38258 | 150.327 |
| 55 | Compound 6442612 | 150.289 |
| 56 | Compound 5318298 | 150.277 |
| 57 | Compound 4417 | 149.899 |
| 58 | Compound 445580 | 149.812 |
| 59 | Compound 5317379 | 149.766 |
| 60 | Compound 5282381 | 149.709 |
| 61 | Compound 442793 | 149.565 |
| 62 | Compound 152128 | 149.425 |
| 63 | Compound 10384247 | 149.305 |
| 64 | Compound 139589863 | 149.22 |

| | | |
|---|---|---|
| 65 | Compound 54685915 | 149.2 |
| 66 | Compound 65130 | 149.178 |
| 67 | Compound 135400182 | 148.871 |
| 68 | Compound 11371027 | 148.625 |
| 69 | Compound 44514059 | 148.292 |
| 70 | Compound 1287 | 148.154 |
| 71 | Compound 10248541 | 148.107 |
| 72 | Compound 688571 | 148.099 |
| 73 | Compound 11198769 | 148.015 |
| 74 | Compound 9869929 | 147.793 |
| 75 | Compound 99307 | 147.773 |
| 76 | Compound 6133 | 147.698 |
| 77 | Compound 76617 | 147.475 |
| 78 | Compound 5282349 | 147.319 |
| 79 | Compound 156580776 | 147.096 |
| 80 | Compound 193112 | 147.087 |
| 81 | Compound 5315472 | 146.973 |
| 82 | Compound 68742556 | 146.705 |
| 83 | Compound 5105 | 146.435 |
| 84 | Compound 77993 | 146.358 |
| 85 | Compound 6440374 | 146.227 |
| 86 | Compound 139590278 | 145.712 |
| 87 | Compound 71765531 | 145.477 |
| 88 | Compound 6101856 | 145.449 |
| 89 | Compound 167846 | 145.214 |
| 90 | Compound 4788 | 145.024 |
| 91 | Compound 145971659 | 145.018 |
| 92 | Compound 44240850 | 144.822 |
| 93 | Compound 168114 | 144.687 |
| 94 | Compound 6072 | 144.582 |
| 95 | Compound 2724635 | 144.517 |
| 96 | Compound 6433266 | 144.486 |

**Table S2.** The evaluation of Lipinski's Rule of Five for 11 compounds.

| ber | MW[1] | LogP[2] | nRot[3] | nHA [4] | nHD[5] |
|---|---|---|---|---|---|
| **nic acid** | 360.08 | 2.005 | 7 | 8 | 5 |
| **in** | 368.13 | 2.147 | 8 | 6 | 2 |
| **chelin** | 374.15 | 1.603 | 10 | 8 | 6 |

| | | | | | |
|---|---|---|---|---|---|
| **linic acid** | 392.15 | 0.96 | 15 | 8 | 1 |
| **5'-triphosphate** | 483.97 | -3.656 | 8 | 17 | 7 |
| **n** | 274.08 | 2.348 | 4 | 5 | 4 |
| **ne triphosphate** | 507 | -3.55 | 8 | 18 | 8 |
| **helin** | 360.13 | 1.329 | 9 | 8 | 6 |
| **elin A** | 404.16 | 1.255 | 11 | 9 | 7 |
| **rol** | 294.18 | 3.199 | 10 | 4 | 2 |
| **acid** | 474.08 | 1.289 | 11 | 12 | 6 |

1. MW: 0-500; **2.** LogP: ≤5; **3.** nRot: 0~11; **4.** nHA: 0~10; **5.** nHD: 0~5.

**Table S3.** The toxicity assessment results of the rosmarinic acid by ADMETLab 3.0**.**

| | Rosmarinic acid |
|---|---|
| hERG blockers | 5.6% |
| Carcinogenicity | 7.2% |
| Eye Corrosion | 1.4% |
| Drug-induced Neurotoxicity | 5.5% |
| Hematotoxicity | 4% |

The data in the table represents the probability of the toxicity occurring.

**Table S4.** Physical properties calculated from two independent MD replicates.

| Parameter | Unit | Replicate 1 (mean ± SD) | | Replicate 2 (mean ± SD) | |
|---|---|---|---|---|---|
| | | PI3K$\alpha$ | PI3K$\alpha$-rosmarinic acid | PI3K$\alpha$ | PI3K$\alpha$-rosmarinic acid |
| RMSD | nm | 0.251 ± 0.0131 | 0.254 ± 0.0176 | 0.243 ± 0.0128 | 0.256 ± 0.0146 |
| RMSF | nm | 0.113 ± 0.0637 | 0.101 ± 0.0455 | 0.125 ± 0.0729 | 0.115 ± 0.0638 |
| SASA | nm² | 571.640 ± 7.2649 | 564.427 ± 6.5671 | 575.388 ± 6.3008 | 576.524 ± 5.8351 |
| Rg | nm | 3.486 ± 0.0083 | 3.479 ± 0.0076 | 3.484 ± 0.0119 | 3.476 ± 0.0088 |
| Hydrogen bond count | count | - | 5.111 ± 1.4041 | - | 2.831 ± 1.0797 |

**Table S5**. The binding free energy (kJ/mol) of PI3K$\alpha$ with rosmarinic acid, and its components between receptor and ligand.

| Energy | PI3K$\alpha$-Rosmarinic acid (Replicate-1) | PI3K$\alpha$-Rosmarinic acid (Replicate-2) |
|---|---|---|
| Van der Waals energy (kJ/mol) | -187.078 | -200.990 |
| Electrostatic energy (kJ/mol) | -178.571 | -94.139 |
| Polar solvation energy (kJ/mol) | 328.739 | 277.668 |
| Nonpolar solvation energy (kJ/mol) | -23.487 | -22.945 |
| Total binding energy (kJ/mol) | -60.397 | -40.406 |
| T$\Delta$S (kJ/mol) | 26.610 | 14.667 |
| Total binding free energy (kJ/mol) | -33.787 | -25.739 |
